# In the Blink of an Eye: Investigating the Role of Awareness in Fear Responding by Measuring the Latency of Startle Potentiation

**DOI:** 10.3390/brainsci2010061

**Published:** 2012-02-16

**Authors:** Ole Åsli, Magne A. Flaten

**Affiliations:** Department of Psychology, University of Tromsø, N-9037 Tromsø, Norway; E-Mail: magne.flaten@uit.no

**Keywords:** startle, fear potentiated startle, delay conditioning, trace conditioning, fear conditioning, contingency awareness

## Abstract

The latency of startle reflex potentiation may shed light on the aware and unaware processes underlying associative learning, especially associative fear learning. We review research suggesting that single-cue delay classical conditioning is independent of awareness of the contingency between the conditioned stimulus (CS) and the unconditioned stimulus (US). Moreover, we discuss research that argues that conditioning independent of awareness has not been proven. Subsequently, three studies from our lab are presented that have investigated the role of awareness in classical conditioning, by measuring the minimum latency from CS onset to observed changes in reflexive behavior. In sum, research using this method shows that startle is potentiated 30 to 100 ms after CS onset following delay conditioning. Following trace fear conditioning, startle is potentiated 1500 ms after CS presentation. These results indicate that the process underlying delay conditioned responding is independent of awareness, and that trace fear conditioned responding is dependent on awareness. Finally, this method of investigating the role of awareness is discussed and future research possibilities are proposed.

## 1. Introduction

### 1.1. Background

In recent decades there has been a debate concerning the role of consciousness and awareness in emotional learning and learning in general. Numerous studies have investigated the effect of awareness in conditioning. In this debate, consciousness is defined as being aware whether the US follows the CS or not, *i.e.*, contingency awareness. Clark and Squire [1,2] argued that in single cue delay conditioning one need not to be aware of the contingency of the CS and US to express a conditioned response. As the association between the CS and the US is made in the cerebellum, for eyeblink conditioning, no cortical areas are needed for this learning to take place. In trace conditioning, however, where there is a temporal gap between the CS and the US, working memory functions would have to contribute to the association. There is not much debate on this second point, however, several researchers argues that unconscious conditioning has not been proven following either form of conditioning (e.g., [3]). 

Numerous attempts have been made to demonstrate unaware conditioning in humans. Clark and Squire [2,4,5,6] investigated whether delay conditioning requires awareness, by studying delay and trace conditioning while manipulating contingency awareness. They concluded that awareness of the CS-US contingency is critical for trace conditioning, but not for delay conditioning. Lovibond and Shanks [3,7], on the other hand, argued that conditioning without contingency awareness has not been proven. They reviewed the literature on awareness in classical conditioning and concluded that almost every study on human classical conditioning used inadequate measures of awareness, and that most measures tend to underestimate awareness, or that the experimental procedures used may lead to underestimated awareness [3,7].

In this review three studies are presented which investigate the latency of the potentiated startle reflex after delay [8,9,10] and trace conditioning [9,10]. By studying startle potentiation at points in time related to CS onset that were too short for controlled processes to contribute, an attempt was made to study unaware conditioning, while bypassing the problems tied to having to manipulate awareness and subsequently measuring awareness by participants self-report. The latency of delay conditioning, and delay and trace fear conditioning, could say something about the amount of processing behind the observed effects of conditioning. In addition, the latency could indicate which structures lay behind these processes. Implications for learning and fear learning are subsequently discussed.

In the following the background is outlined, with emphasis on fear, the startle reflex, conditioning and awareness. This is followed by a brief presentation of three empirical studies and a general discussion, before an attempt is made to generalize the implications of the studies to the world outside the laboratory. 

### 1.2. Consciousness

In the context of conditioning, *conscious* may be defined as a concept that characterizes awareness or knowing, that pertains to the process of being aware, or it characterizes a reaction of which the individual is aware. Similarly, it can point to that which is part of the perceptual conscious, *i.e.*, the portion of mind aware of the immediate environment [11]. In light of the research on emotions and especially fear conditioning the focus will be on the concepts of automatic processing, awareness and contingency awareness. 

#### 1.2.1. Automatic Processing and Awareness

Automatic processing, or pre-attentive information processing, refers to the registration, analysis and identification of a stimulus. These processes occur as a response to the stimulus, whether voluntary attention has been directed to the stimulus or not [12]. Automatic is commonly interpreted as independence from top-down factors, such as attention and task instructions. The term “pre-attentive” indicates that the initial processing of the stimulus is performed prior to conscious identification of the stimulus, and several studies have indicated that subliminally presented stimuli affect behavior and physiology. As noted by Moors and De Houwer [13] awareness is a feature of automaticity and relates to the participants perception of presented stimuli. When the participant reports not being conscious of a briefly presented stimulus, he or she is said to be unaware. If, on the other hand, the participant reports to have seen or heard, or in any other way sensed the stimulus, the participant is held to be aware. In addition to such subjective criteria of awareness, it is common to employ more objective criteria, e.g., that participants are labeled unaware when they perform at chance in a forced-choice task [14]. On a related note, lack of awareness of a process precludes controlling that process [13], underlining the close link between the concept of awareness and automaticity.

#### 1.2.2. Problems Related to Measuring Awareness

The measurement of awareness is a topic for much discussion (e.g., [3,7,15]). For the present review, the subject of contingency awareness is the key construct when it comes to the different understandings of awareness. Lovibond and Shanks [3] criticized almost every study on the role of consciousness in classical conditioning, on the grounds that the method used to measures awareness, tend to underestimate awareness or that the experimental procedures used may lead to underestimated awareness. As Lovibond and Shanks [3] point out, there are substantial problems related to measuring awareness by participant’s self-report. These are problems which will be discussed in more detail later in this review.

### 1.3. Fear

Do we feel afraid because we tremble, or do we tremble because we are afraid? This has been one of the most important questions in emotion and fear research since James [16] argued that the former was the case. This somewhat counterintuitive statement relates to the important question about whether fear is a cognitive and controlled process or an automatic pre-conscious one. This is still a heavily discussed topic (e.g., [3,7,14,17,18,19,20]). But first; what is fear? 

Fear is evoked by threatening, intense, novel and sudden stimuli. The reaction to these stimuli involves autonomic, somatic and subjective responses, and is often termed a fight-or-flight response. In the first physiological theory of emotion, by William James and the Danish physiologist Carl Lange, the perception of the autonomic and somatic reactions resulted in the experience of an emotion. As such, fear would be the brain’s perception of the fight or flight response. Later, this view was challenged by the Cannon-Bard theory, which stated that emotional experience and emotional expression are parallel processes [21]. Their view held that fear stimuli would have two separate effects: they would excite the feeling of fear in the cortex and the expression of emotion in the autonomic and somatic nervous systems. 

#### 1.3.1. Fear in the Brain

Much of what is known about the relation between learning, fear and the corresponding brain functions comes from animal research. Animals lack the ability to verbally communicate their experiences; however, they have several features of the defensive system in common with humans. Among these are defensive behaviors such as startle and fear-potentiated startle. The way basic emotional systems are organized in the brain seems to be similar across species, and there seems to be small differences within the group of animals who has a brain and a backbone [17]. Accordingly, models of animal defensive behaviors have been used as models for human fear as well [18]. These models points to subcortical structures and highlights the amygdala as a sort of emotional computer. 

##### 1.3.1.1. The Amygdala

Animal studies have shown that amygdala lesions disrupt conditioned fear, even when conditioned stimuli in different sensory modalities are used [22]. Human studies on the topic have underlined the significance of the amygdala in human fear. Human stimulation and lesion studies, PET recordings [23,24], and fMRI recordings [25,26,27] support the idea that the amygdala is centrally involved in fear. It is also found that the amygdala is central in recognizing facial expression of fear [28,29,30]. Large numbers of benzodiazepine receptors are found in the amygdala (e.g., [31]). Benzodiazepines are major tranquilizers used to reduce anxiety, and infusion of benzodiazepines in the central nucleus abolishes the fear reaction. Thus, this finding supports a role for the amygdala in fear and anxiety. 

The amygdala seems especially important in learning and remembering fear. Two early studies on amygdala function in human fear conditioning found impaired fear conditioning in patients with amygdala damage [32,33]. Both studies found that the patients showed normal URs to the US, but they failed to acquire a normal fear response to the CS. However, studies on humans with amygdala damage do not have the same accuracy and flexibility that animal lesion studies have. 

A recent study by Antoniadis, Winslow, Davis and Amaral [34] showed that lesions of the amygdaloid complex in rhesus monkeys blocked the acquisition of fear-potentiated startle. This is in agreement with previous research in rats [35,36]. However, animals that sustained amygdala damage after they successfully learned fear potentiated startle, expressed normal fear potentiated startle [34]. This suggests that memory of fear-potentiated startle may be stored in extra-amygdala areas and that this memory is sufficient for the expression of fear potentiated startle. Thus, although the research on rodents is important, it is not clear to what extent this research can be extrapolated to primates and, consequently, more research in primates is needed. 

##### 1.3.1.2. The Pathways to the Amygdala

Studies in rats have revealed two pathways to the amygdala for fear signals. Conditioning to a single tone paired with footshock involves transmission to the auditory relay nucleus in the thalamus [37]. From the auditory thalamus the signal is transmitted to the auditory cortex, and from a subset of thalamic nuclei to the amygdala. This direct pathway from the thalamus to the lateral nuclei of the amygdala is termed the thalamo-amygdala pathway and is thought to be a “quick and dirty” route for the fear signal. From the lateral nucleus the information is relayed to the central nucleus [37]. This could be the route via which unconscious or pre-attentive input reaches the amygdala [28]. The auditory association cortex also gives rise to a projection to the amygdala [38] called the thalamo-cortico-amygdala pathway. This is thought to be a slower route for the fear signal, but carrying more detailed information of the fine-grained analysis of the fear relevant stimuli occurring in the cortex. The results of our research are compatible with the idea that these two pathways are in operation also in humans. 

#### 1.3.2. Pre-Conscious Fear

Do humans have a system for quick, pre-conscious fear processing? Numerous studies have contributed to answering this question. Studies by Öhman and collaborators [39] have indicated that pictures with fear-relevant content can increase sympathetic activation, such as skin conductance responses (SCRs), even when the pictures are presented subliminally. Fear-relevant stimuli can be pictures of angry faces, snakes, spiders, *etc.* However, in Öhman’s experiments, the fear reaction only emerges when the fear-relevant pictures have been paired with an aversive unconditioned stimulus. Since the fear-relevant pictures are associated with an aversive event, the conditioned arousal is interpreted as a sign of conditioned fear. Subliminally presented conditioned fear-irrelevant stimuli (pictures of, e.g., flowers or mushrooms) do not increase sympathetic activation. Öhman’s experiments have shown that participants show increased SCRs even when the feared stimulus is subliminally presented. It is hypothesized that this is a conditioned fear reaction and that the subliminally presented conditioned stimulus activates the amygdala, via thalamic or early cortical processing regions, with the consequent elicitation of a fear response [23].

Recent studies have found that initially fear-irrelevant stimuli also may elicit fear without cognitive controlled processing. Hamm *et al.* [40] showed acquisition of fear-conditioned startle potentiation to an initially neutral visual CS in a patient suffering from bilateral cortical blindness. Despite not being able to process the conditioned stimuli in the primary visual cortex, the patient reacted with potentiated startle to the CS. In a similar vein of research, Knight, Nguyen and Bandettini [41,42] found conditioned SCR discrimination to tone CSs presented at subthreshold intensities. However, this SCR discrimination appeared only following a delay conditioning procedure and not a trace conditioning procedure, an important difference which will be discussed later. 

Taken together, these findings are interpreted as evidence that we are equipped with inborn tendencies to respond to fear-related stimuli [17,19]. Therefore, fear-related stimuli enter easily into associations with aversive events, even when the fear-related stimulus is subliminally presented. 

### 1.4. The Startle Reflex

The startle reflex is described by its eliciting stimulus, the physiological and behavioral reaction to the stimulus, and the neural mechanism that mediates the reaction.

The prime characteristic of stimuli that elicit startle is their sudden onset. Auditory stimuli with rise-times of less than 10 ms are typically used to elicit startle. That is, the maximum sound pressure level is reached in less than 10 ms from stimulus onset. However, there is a trade-off between suddenness and intensity of the stimulus, so more intense stimuli can have slower rise-times, but still elicit startle. With close to instantaneous rise-times, startle can be elicited by auditory stimuli with intensities of only 60 dB [43]. Startle can also be elicited by tactile and visual stimuli [44]. 

The startle reaction consists of a series of muscular activities that involve eyeblinks and contraction of muscles in the neck, shoulders, upper back, arms and legs. The blink reflex consists of the coordinated movements of several muscle groups, primarily contractions of the orbicularis oculi, which has fibers in the eyelid and around the eye, and reciprocal inhibition of the levator palpebrae, which raises the eyelid. Activity in the orbicularis oculi is detected with electromyographic (EMG) methods and is the most used index of startle in humans. Lid closure occurs about 10–12 ms after the initial orbicularis oculi EMG activity [45]. 

#### 1.4.1. Startle Reflex Modification

The amplitude, latency and probability of the startle reflex can be modified by psychological processes, termed startle reflex modification. One of the most studied forms of startle reflex modification is called pre-pulse inhibition (PPI). PPI appears as startle is inhibited by weak stimuli presented immediately prior to the startle reflex-eliciting stimulus. The reason for the inhibition is probably that the first, weak stimulus initiates automatic processing, and subsequently presented stimuli receive less processing as long as the first stimulus is being analyzed [46,47]. Thus, the startle-eliciting stimulus receives less processing and, therefore, the reflex is inhibited. This process lasts for about 400 ms [48,49,50], and is used in human and animal studies as an index of automatic processing, *i.e.*, processing that occurs immediately and involuntarily to novel stimuli, before controlled attention is directed (or not) to the stimulus. It is, thus, assumed that PPI is an index of automatic processing. Empirical studies have supported this hypothesis. Norris and Blumenthal [51] found that subjects who reported correctly the frequency of auditory and tactile stimuli, and for that reason probably paid close attention to those stimuli, displayed increased PPI compared to subjects who did worse on that task. Similar findings have been obtained by Elden and Flaten [52]. Elden and Flaten [48] found increased PPI when subjects were asked to pay attention to the weak stimulus. PPI seems not to be affected by arousal, as administration of an arousal-inducing substance, caffeine, had no effect on PPI [53]. 

#### 1.4.2. Startle as a Measure of Fear: Startle Reflex Potentiation

The startle reflex is modulated by other psychological processes as well, most notably by fear. Lang and colleagues [54] have shown that the startle reflex is affected by the emotional state of the organism, with larger reflexes when the organism is in a defensive state, and smaller reflexes in a pleasant state. Increased startle amplitude when the subject is in a state of fear is called *fear potentiated startle*. Fear potentiated startle seems to be less affected by fear irrelevant arousal [55]. In addition, stimuli inducing positive emotions inhibit startle, which means that startle may be used as a measure of affective valence, dissociating between positive and negative emotions, while skin conductance reflects arousal [54]. The difference between valence and arousal is important. Arousal is general in nature, related to the intensity of affect, and may be due to positive or negative emotional ques. Valence, on the other hand, is tied to the quality of the emotion, related to a continuum from pleasant to unpleasant. As such, emotions can be characterized by two dimensions: valence (pleasant-unpleasant) and arousal (high-low). 

Fear potentiated startle, then, has the opposite effect compared to PPI, and both fear and attention may affect startle. That is, a tone preceding the startle eliciting noise lead to a decrease in startle reflex amplitude at short stimulus onset asynchronies (SOA) from about 15 to 400 ms [49,50]. However, a tone could also be the conditioned stimulus signaling an upcoming US in participants who has received classical conditioning. As such, the CS tone potentially facilitates startle reflex amplitude, because of fear or other processes related to expectance of the US. Consequently, the total startle reflex amplitude would be a summation of the inhibiting effect of PPI and the facilitating effect of potentiation. As a result, potentiated startle would not always be facilitated compared to startle amplitudes to trials with startle eliciting noise alone, however, compared to conditions where only PPI influence the reflex, potentiation is expected. 

The studies reported here measure the latency of startle reflex potentiation. The latency of the startle reflex *per se* is not a topic in the present review. It is the time from presentation of the CS to potentiation of the startle reflex can be observed that is of interest in the three studies reported here. 

Attention may also modulate PPI and consequently, the startle reflex. The typical finding for attentional modulation is reliably greater PPI to attended stimuli than ignored stimuli at a 120 ms SOA and no reliable differences at shorter intervals [56,57]. There is also at least one study that has shown attentional modulation at a lead interval as early as 60 ms [58]. This is an account of early startle modulation which could complicate the interpretation of the influence of fear on startle at similar short intervals. However, attention increases PPI, or decreases startle, the opposite of the effect of fear on startle. Thus, if potentiated startle is found at short SOAs this effect cannot be mistaken for attentional effects of startle. However, it is possible that attention could decrease the effect of fear on startle. 

#### 1.4.3. Measuring the Latency of the Fear Reaction

When attempting to measure the latency of the fear reaction, a measure of fear that is as precise as possible is needed. Previous studies have used skin conductance responses to masked conditioned fear-evoking stimuli [23,59]. The advantage of the fear potentiated startle method is that it (1) is a measure of affective valence and not general arousal [54,55]. (2) It provides significantly better temporal resolution than SCRs. This allows the investigation of the speed and development of the fear reaction, as well as its intensity. (3) The neural basis of the startle reflex is well known from animal and human studies. (4) The neural basis of fear-potentiated startle is partly known from animal studies.

### 1.5. Classical Conditioning

#### 1.5.1. Awareness in Conditioning

In classical conditioning awareness may reflect two factors. In most experiments both the CS and US are presented in such a way that they are easily detected. However, when the CS and/or US are not easily detected, awareness may be assessed by criteria mentioned in 1.2.2. If the stimuli are perceived by the participant, another form of awareness, contingency awareness, comes in to play. Participants are said to be contingency aware when they correctly report the relationship between the CS and the US. In most studies involving classical conditioning, the term awareness is coined to reflect contingency awareness. The term consciousness has been used more loosely. 

#### 1.5.2. Delay and Trace Conditioning

In delay conditioning the CS and the US overlaps. Several studies have reported that delay conditioning may occur without contingency awareness (e.g., [1,2,32,60,61]). The reasoning behind this is that the association between the CS and the US is formed subcortically, e.g., in the cerebellum for eyeblink conditioning [62,63,64], and that the process is independent of the hippocampus [65,66]. 

In trace conditioning there is a time gap between the CS and US. This small procedural difference has some important consequences. Trace conditioning is dependent on hippocampus involvement [67], whereas delay conditioning is not [68], because the hippocampus is needed to associate two stimuli separated in time. Several studies have shown that the participants must be aware of the CS-US contingency for trace conditioning to have effect [1,2,61].Thus, the debate is whether delay conditioning may be formed without contingency awareness or not. Despite numerous studies stating the former, Lovibond and Shanks [3,7] argued that such unconscious conditioning has not been proven. They declared that the reviewed studies on the role of consciousness in delay classical conditioning used inadequate measures of awareness, underestimated awareness or used experimental procedures that led to underestimated awareness. 

#### 1.5.3. Temporal Specificity of Fear Conditioning

Davis *et al.* [69] investigated fear potentiated startle in rats as a function of interstimulus interval duration in fear conditioning. Different groups received fear conditioning with interstimulus intervals ranging from 0 to 51.2 s. Fear potentiated startle was tested at intervals from 50 ms to 100 s after CS onset. The results showed that potentiated startle was at maximum at, or close to, the interval used in training. The accuracy of the temporal specificity increased with increasing number of conditioning trials, but temporal specificity could be seen after one single trial when the 51.2 s CS-US interval had been used in training. 

The results from Davis *et al.* [69] indicate that to observe the minimum latency of the fear reaction, a short interstimulus interval should be used in training. Furthermore, significant fear potentiated startle can be observed at shorter SOAs than the interstimulus interval used in training. However, potentiation decreases as the time for testing startle potentiation diverge from the time were the US was presented in relation to the CS during training.

### 1.6. The General Idea

The next section will describe three studies using startle potentiation at different SOAs to investigate the role of awareness in fear responding. The basic idea is as follows: if startle is potentiated at short SOAs following the feared conditioned stimulus; this would imply that the underlying process is automatic and unaware. However, should startle only be potentiated at longer lead intervals, this would point towards a controlled underlying process. Is this simple logic viable? That is an important question in which we will return to in the *General Discussion* section.

## 2. Investigating the Role of Awareness by Measuring the Latency of Startle Potentiation

### 2.1. Methods

The methods used in all the three studies are similar. All three studies were classical conditioning studies with a tone CS. The US was an airpuff to the corner of the eye in Åsli and Flaten [8], white noise in Åsli, Kulvedrøsten, Solbakken and Flaten [10], and electrical stimulation of two fingertips in Åsli and Flaten [9]. In general the studies consisted of two phases. The conditioning phase; where the tone CS was paired with the US in the paired group, and presented in an explicitly unpaired manner in the unpaired group. The startle phase: different stimulus onset asynchronies (SOA) between the CS and US varied from study to study but always consisted of short intervals (150 ms and less) and longer intervals (more than 150 ms). 

Davis *et al.* [69] showed that fear potentiated startle can be observed at shorter SOAs than the interstimulus interval used in training. Furthermore, that study revealed that an interstimulus interval of 800 ms resulted in potentiated startle at 50 to about 6000 ms and that the potentiation was at maximum at the 100 ms SOA [69]. Consequently, an interstimulus interval of 1000 ms as used in Åsli *et al.* [10] and Åsli and Flaten [9] should potentiate startle at both early and late SOAs.

In addition to the startle reflex, subjective measures were also included in all studies. In Åsli and Flaten [8] emotional valence and arousal elicited by the CS and the airpuff US was assessed with the Self Assessment Manikin (SAM) [70]. In Åsli *et al.* [10] and Åsli and Flaten [9] emotional valence was recorded with a 10 cm visual analog scale (VAS) asking the participants to rate the CS and US on an unpleasant-pleasant-continuum. In Åsli *et al.* [10] nervousness elicited by the CS was also recorded on a VAS in a similar manner. In Åsli *et al.* [10] and Åsli and Flaten [9], CS-US-contingency awareness was assessed by a VAS containing three statements recording the participant's understanding of the relationship between the CS and US. There was one difference between the two studies in how the contingency-VAS was administrated. In Åsli *et al.* [10] the VAS was given after the startle phase. However, in Åsli and Flaten [9] the VAS was administrated directly after the conditioning phases (and after the startle phases), to ensure that the awareness assessment was as close to the conditioning phase as possible.

### 2.2. Study 1: Conditioned Facilitation of the Unconditioned Reflex After Classical Eyeblink Conditioning [8]

#### 2.2.1. Structure

**Figure 1:**
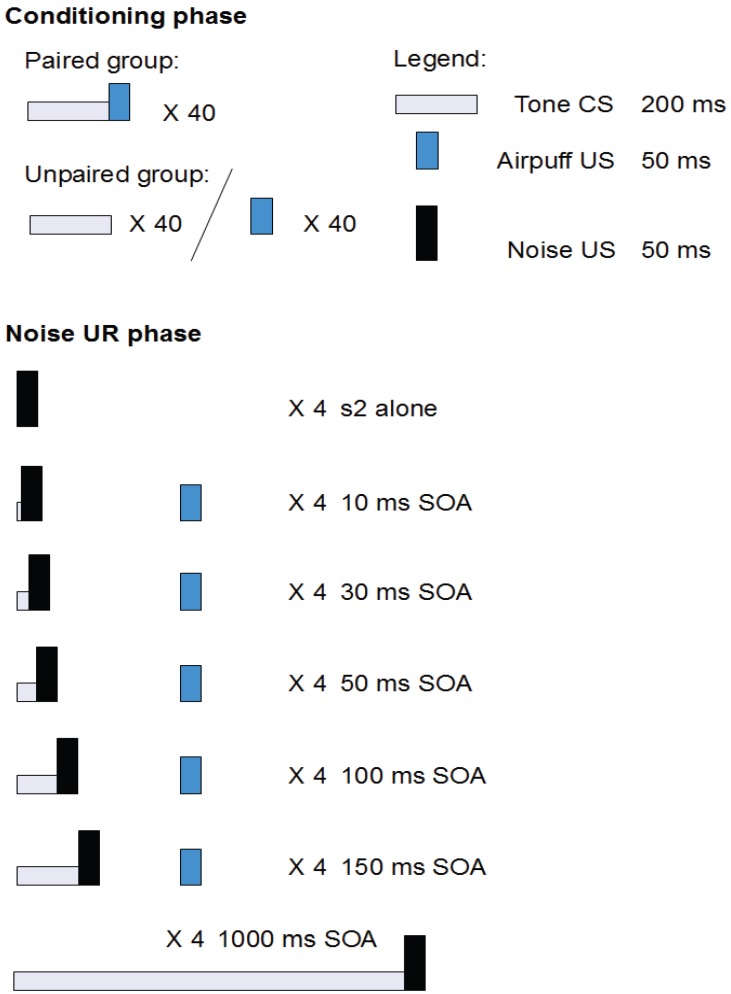
A schematic presentation of the experiment structure.

The experiment consisted of two phases (see Figure 1): In the conditioning phase, the paired group received 40 trials of single-cue classical conditioning and the unpaired group received 40 explicitly unpaired presentations of the CS and the US. In the noise UR phase, UR facilitation to the noise US was investigated by presenting the CS at various lead intervals relative to the onset of the eyeblink eliciting noise.

#### 2.2.2. Aim and Background

The study investigated the time course of conditioned facilitation of the unconditioned eyeblink reflex. Several studies have shown that unconditioned reflexes (URs), elicited in the presence of a conditioned stimuli, are potentiated compared to URs elicited in the absence of the CS [71,72,73,74]. Such potentiation of the UR is thought to index an early stage of eyeblink CR formation [71,73,74]. The process underlying the UR potentiation could be CS-induced facilitation of the motoneuron pool in the facial nerves responsible for the eyeblink (e.g., [75]). Thus, when a startle eliciting stimulus is presented to an already facilitated motoneuron pool, the magnitude of the reflex increases. 

The role of awareness in classical conditioning is debated. Clark and Squire [76] argued that single-cue delay classical conditioning is independent of awareness of the CS-US-contingency. However, Lovibond and Shanks [3] argued that conditioning independent of awareness has not been proven. 

Åsli and Flaten [8] aimed to bypass the problems tied to measuring awareness in classical conditioning by investigating UR potentiation at points in time related to CS onset that were too short for conscious processes to play a role. 

#### 2.2.3. Results

**Figure 2:**
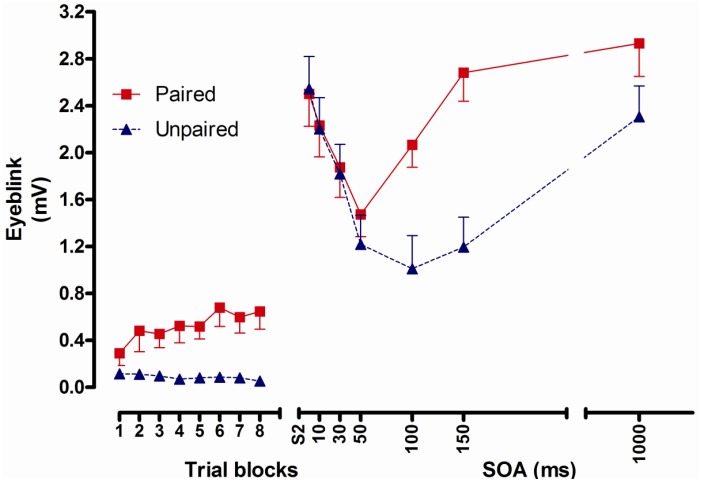
Left panel: Conditioned eyeblink responses in the paired and unpaired groups in the conditioning phase. Error bars represent 1 standard error of the mean. Right panel: Noise UR magnitudes across SOAs. S2 denote noise alone trials.

The analysis of CR responding revealed a significant main effect of conditioning, due to significantly greater conditioned responses in the paired group compared to the unpaired group. There was also a significant interaction of conditioning by trial block, and follow-up tests showed significantly larger CRs in the paired group compared to the unpaired group in trial blocks 3–8 (Figure 2). 

Analyses of unconditioned reflex potentiation showed a significant interaction of conditioning by SOA, and follow-up tests revealed significantly potentiated URs elicited 100 and 150 ms after CS onset in the paired group compared to the unpaired group (Figure 2). 

Correlational analyses showed a significant positive correlation between CRs in the second half of the trials in the conditioning phase, and noise URs at the 100, 150 and 1000 ms SOA in the noise UR phase. 

#### 2.2.4. Conclusions

Åsli and Flaten [8] showed reflex potentiation as early as 100 ms after CS presentation in the paired compared to the unpaired group. This reflex potentiation was related to CR magnitude, indicating that the CR was mediated by an automatic and pre-attentive process. This is in agreement with previous studies showing that delay conditioning may occur without awareness, and potentiation at short SOAs may be a method to study this without assessing contingency awareness with error prone self-reports. 

### 2.3. Study 2: Fear Potentiated Startle at Short Intervals Following Conditioned Stimulus Onset During Delay but Not Trace Conditioning [10]

#### 2.3.1. Structure

**Figure 3:**
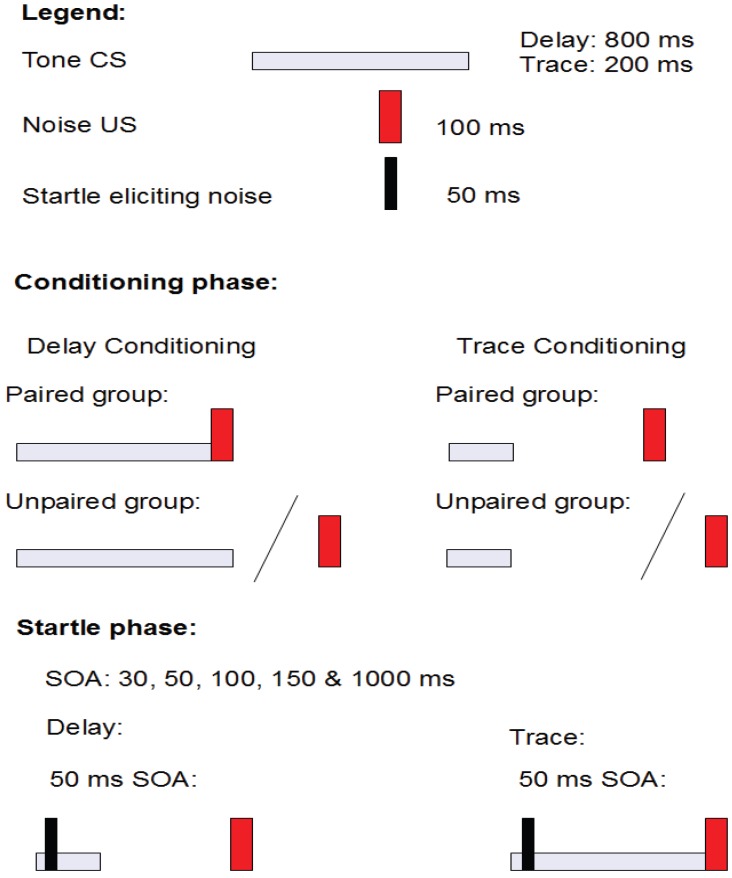
A schematic presentation of the structure of the experiment in Åsli *et al.* [10].

The experiment consisted of a conditioning phase and a startle phase (see Figure 3). In the conditioning phase, the paired groups (delay and trace) received 14 trials of single-cue classical conditioning, and the unpaired groups received 14 explicitly unpaired presentations of the CS and the US. In the startle phase, fear potentiated startle was tested by presenting the startle eliciting noise at various lead intervals relative to the CS onset.

#### 2.3.2. Aim and Background

In Åsli *et al.* [10], the latency of the conditioned fear reaction, following delay and trace conditioning, was investigated with aversive noise as the US. Trace conditioning, where there is a temporal gap between the CS and US, is said to be dependent of the hippocampus, whereas delay conditioning is not [60,61,76]. Accordingly, adding a trace condition made it possible to investigate the difference in latency of the conditioned fear reaction between a quick hippocampus independent route of fear learning and a slower hippocampus dependent route. 

This would shed further light on the debate concerning awareness in conditioning. If one could find potentiated startle at shorter intervals of the tone CS in delay conditioning than trace conditioning, this would be in line with research showing that delay conditioning could occur without awareness whereas trace conditioning could not. 

#### 2.3.3. Results

**Figure 4:**
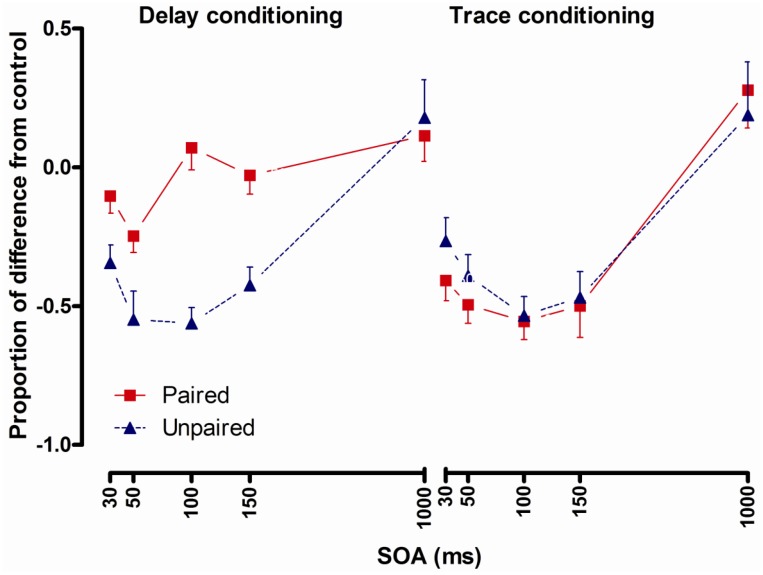
Startle reflexes following delay conditioning (left panel) and trace conditioning (right panel) expressed as proportion of difference from control in the paired and the unpaired group across stimulus onset asynchronies (SOA). Error bars represent 1 standard error of the mean.

The interaction of type (delay, trace) by conditioning (paired, unpaired) by SOA was significant and contrast analyses showed significantly increased startle in the delay paired group compared to the delay unpaired group at the 30, 50, 100 and 150 ms SOA (Figure 4). There was no significant difference between the trace paired and the trace-unpaired group at any SOA. Contrast analyses showed significantly increased startle in the delay paired compared to the trace-paired group at the 30, 50, 100 and 150 ms SOA. There were no significant differences between the delay unpaired and the trace unpaired group. 

The CS-US-contingency data revealed no significant difference between the delay paired and the trace paired, or between the delay unpaired and the trace-unpaired groups. There was a significant main effect of conditioning due to more correct answers to the awareness questions in the paired groups compared to the unpaired groups. 

#### 2.3.4. Conclusions

Åsli *et al.* [10] showed a fear reaction only 30 ms post stimulus onset following delay conditioning, as evident by the potentiated startle in the paired group compared to the unpaired group. Startle was also potentiated at short SOAs in the delay-paired group compared to the trace-paired group. There was no difference between trace and delay conditioning when it comes to awareness of the CS-US-contingency, indicating that the participants understood the relationship between the CS and the US equally well regardless of type of conditioning. The potentiated startle at such short intervals following CS onset during delay conditioning, but not trace conditioning, could indicate that the two types of conditioning are supported by different structures. This is line with the notion that delay conditioned responding may occur without awareness, but trace conditioned responding may not. 

### 2.4. Study 3: How Fast Is Fear? Automatic and Controlled Processing in Conditioned Fear [9]

#### 2.4.1. Structure

**Figure 5:**
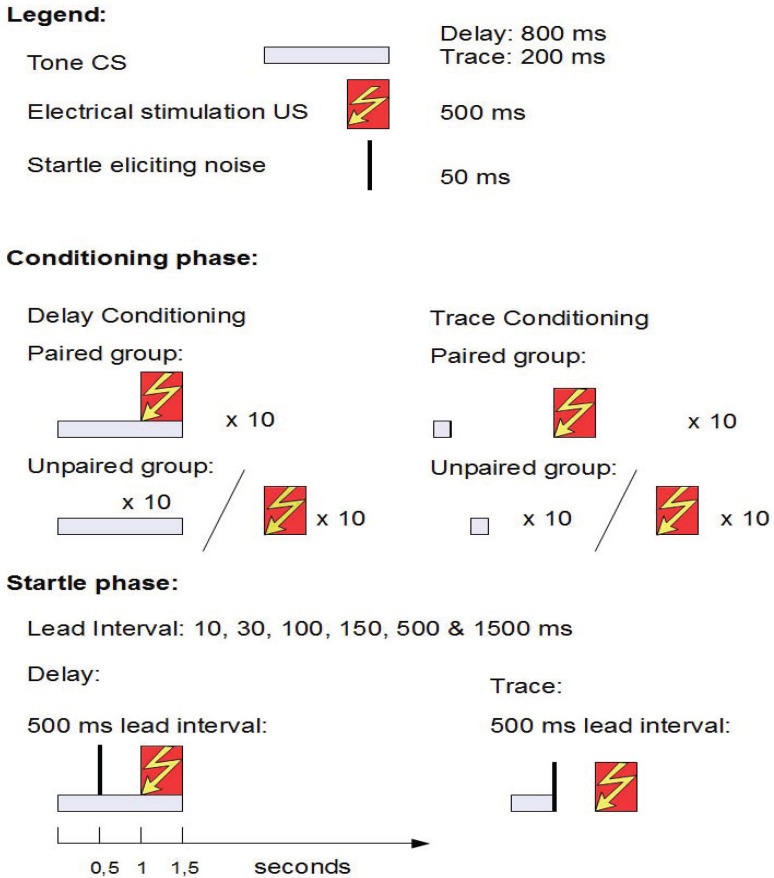
A schematic presentation of the structure of experiment the experiment in [9].

The experiment consisted of a conditioning phase and a startle phase (see Figure 5). In the conditioning phase, the paired groups (delay and trace) received 10 trials of single-cue classical conditioning, and the unpaired groups received 10 explicitly unpaired presentations of the CS and the US. In the startle phase, fear potentiated startle was tested by presenting the startle eliciting noise at various lead intervals relative to the CS onset. 

#### 2.4.2. Aim and Background

In Åsli and Flaten [9], the latency of the conditioned fear reaction, following delay and trace conditioning, was investigated with electrical shock as the US. This was a methodological improvement compared to Åsli *et al.* [10]. In Åsli *et al.* [10] an intense noise was used as the US, and eyeblink CR could have contributed to the startle reflexes in the paired groups, and been mistakenly interpreted as evidence of fear potentiated startle. In addition was a 1500 ms SOA included to increase the likelihood of obtaining potentiated startle in the trace-paired group. A third improvement was the contingency awareness test was administered directly after the conditioning phase to keep interference and forgetting to a minimum.

#### 2.4.3. Results

**Figure 6:**
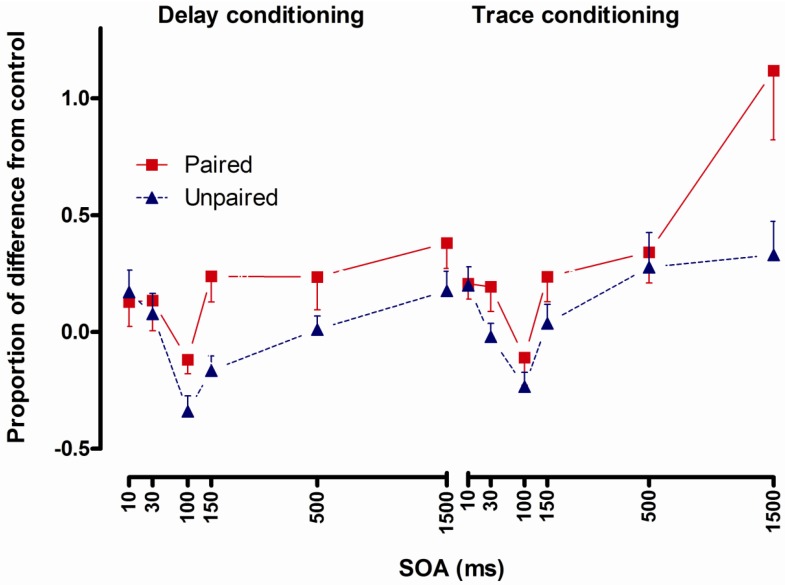
Startle reflexes following delay conditioning (left panel) and trace conditioning (right panel) expressed as proportion of difference from control in the paired and the unpaired group across stimulus onset asynchronies (SOA). Error bars represent 1 standard error of the mean.

The interaction of Type by Conditioning by SOA was significant and contrast analyses showed increased startle in the delay paired group compared to the delay unpaired group 100 and 150 ms post CS onset and the trace paired group showed increased startle at 1500 ms after CS onset compared to the trace unpaired group (Figure 6).

#### 2.4.4. Conclusions

Two main findings emerged in Åsli and Flaten [9]: Startle was potentiated in the delay-paired group compared to the delay-unpaired group at the 100 and 150 ms SOA, and startle was potentiated in the trace-paired group compared to the trace-unpaired group at the 1500 ms SOA. Startle potentiation at short SOAs in the delay-paired group provides further indication that the underlying process is automatic and preconscious. Potentiated startle in the trace paired group at the 1500 ms SOA, and not at shorter SOAs, indicates that trace fear conditioned responding is due to controlled processes. 

## 3. General Discussion

The main findings from this research were as follows: Startle was potentiated in the delay paired group compared to the delay unpaired group at short intervals after CS onset in all three studies: at the 100 and 150 ms SOA in Åsli and Flaten [8,9] and at the 30, 50, 100 and 150 ms SOA in Åsli *et al.* [10]. In addition, there was potentiated startle in the trace-paired group compared to the trace-unpaired group at the 1500 ms SOA in Åsli and Flaten [9]. Startle potentiation at short SOAs in the delay-paired group indicates that the underlying process is automatic and preconscious. Potentiated startle in the trace paired group at the 1500 ms SOA, and not at shorter SOAs, indicates that trace fear conditioned responding is due to controlled processes. 

Åsli *et al.* [10] and Åsli and Flaten [9] revealed no effect of trace fear conditioning at the SOAs from 10 to 500 ms. These two studies also showed startle potentiation during delay conditioning at intervals from 30 to 150 ms after CS onset in Åsli *et al.* [10] and at the 100 and 150 ms interval in Åsli and Flaten [9]. The only effect of conditioned fear following trace conditioning was found 1500 ms after CS onset in Åsli and Flaten [9]. Intuitively one could think that even tough learning of trace conditioning is mediated by controlled processes; once the learning is formed the access to the learned information should not be different compared to delay conditioning. 

Clark and Squire [2] argued that one must also be able to access the learned information about the CS-US contingencies during the condition session itself in order to express learning acquired from trace conditioning. The results from the Åsli *et al.* [10] and Åsli and Flaten [9] fit well with this notion. Clark and Squire [2] proposed that trace conditioning is dependent on the hippocampus because the trace interval between the CS and the US makes it difficult to process the CS-US relationship in an automatic, reflexive way. In delay conditioning, because the CS is present during presentation of the US, the amygdala, for fear conditioning, has access to the CS and US information simultaneously. For trace conditioning, more processing of the CS is needed to form the memory of the CS-US contingency because of the gap between the CS and the US. If the stimulus contingencies are represented in the neocortex, the processed information about the CS-US contingency may be sent to the amygdala. The longer latency of trace fear conditioning indicates that one has to access the CS-US contingency information each time the CS is presented and, accordingly, the transmission of this extra information could be the reason why the latency of the CR is longer for trace conditioning than delay conditioning. 

This proposed difference in information processing between delay and trace conditioning is in line with research which has revealed that there are two different pathways for the fear signal to reach the amygdala [77,78]. The fear signal, *i.e.*, the CS, may travel from the medial geniculate nucleus of the thalamus to the dorsal lateral nucleus of the amygdala. If the information about the CS travels via the thalamo-amygdala pathway, this could be sufficient to activate the amygdala following delay conditioning, as the CS-US association is presented in the amygdala. If the CS signal following trace conditioning reaches the amygdala through the thalamo-amygdala pathway, there would be no information stored about the contingency with the US in the amygdala. However, the CS signal could also travel via the other pathway for fear signals, which is the thalamo-cortico-amygdala pathway where the fear signal is processed in cortical areas before reaching the amygdala. The CS-US contingency information could be stored there and subsequently added to the signal, and then activate the amygdala. Accordingly, as the fear content of the CS is probably activated only via the longer and slower thalamo-cortico-amygdala pathway following trace conditioning, this could be the explanation for the longer latency observed following trace conditioning. 

The participants in the reviewed studies were all aware of the contingency between the CS and the US. Could this awareness have influenced the participants’ responding even at the shortest SOAs? It is possible that the expectancy of an aversive stimulus could have increased the startle response as a consequence of establishing the contingency between the CS and the US. In other words, a cognitive process such as contingency awareness could, if not trigger the fast startle potentiation, still be an influential factor in the process. In fact, a recent study by Lovibond *et al.* [79] has shown a clear link between awareness and CR responding in a differential conditioning study with both delay and trace conditioning. These data suggest that both delay and trace conditioning depend on a neural system that involve conscious and controlled processing as proposed in the single-system model of learning by Mitchell *et al.* [80]. However, the study by Lovibond, Liu, Weidemann and Mitchell [79] deployed a differential conditioning procedure, a more complex procedure than the single cue conditioning procedure used in the reviewed studies. It could be that the process of distinguishing between the two auditory CSs made the process dependent on contingency awareness. In addition, in the reviewed studies, contingency awareness did not differ in the delay and trace paired groups. Consequently, if contingency awareness influenced the process of potentiation at the shortest intervals in delay conditioning, why did not the same thing happen in trace conditioning in study 3? Åsli and Flaten [9] found startle potentiation after both delay and trace conditioning, in participants who were all contingency aware, the only difference being that startle potentiation occurred at short intervals following delay conditioning and only at longer intervals following trace conditioning. In the absence of a better explanation for this effect, the most salient explanation, in our opinion, is that the faster fear reaction following delay conditioning was due to different underlying processes in the two conditioning procedures. Recent neuroimaging studies support this reasoning. Tabbert *et al.* [81] found conditioned neural responses in contingency aware participants, but also in contingency unaware participants in a conditioning study using visual conditioned stimuli and distracters. This report is consistent with earlier research from the same group [82,83] confirming the independence of conditioned amygdala responses from contingency awareness.

Åsli and Flaten [8], Åsli *et al.* [10] and Åsli and Flaten [9] show that startle potentiation at short intervals following CS onset can be applied as a method for assessing automatic, unaware associative responding. All three studies show startle potentiation at 100 ms or shorter after CS onset. This interval is too short for any contribution from controlled processes [56,57]. Åsli and Flaten [9] show that CRs generated by controlled processes, such as in trace conditioning, has a latency between 500 ms and 1500 ms. Furthermore, it may be possible to investigate if other learning processes are mediated by controlled or automatic process with the tool of startle potentiation at short intervals following CS onset. For instance, this method could be used to determine if differential conditioning is mediated by controlled processes. As such, one would expect startle potentiation to, e.g., two different tone CSs at short intervals following CS onset, even though only one of them, the CS+, has been followed by the US. At longer intervals only the CS+ should be potentiated as the controlled process of differentiation of the two tones has come into play. The CS−, which is not followed by the US, should cause no potentiation. An experiment by Weike, Schupp and Hamm [84] has indicated such a time specific effect of differential conditioning as they found fear potentiated startle to both a CS− and CS+ at early probe times after CS onset, but only after CS+ at longer intervals. This study, however, was not designed to investigate the effect of early *versus* late probe times of differential conditioning. Other learning processes which could be studied with this method in regard to whether they are mediated by automatic or controlled process include, but are not limited to; extinction, instructed fear and observational learning.

The results from Åsli *et al.* [10] and Åsli and Flaten [9] could also indicate that fear responding may occur without conscious awareness of the contingency between the CS and US. This means that one could experience a fear reaction without being aware of what elicited the reaction. Such an unspecific fear is a component of several fear and anxiety disorders, such as panic attacks and generalized panic disorders. There are at least three important implications of this. First of all, this means that the brain knows more than can be verbally expressed. We may have no idea why we react with fear in some circumstances, but this information is nevertheless stored in our brains. Second, learning which is similar to delay fear conditioning could be the cause of at least some instances of, e.g., fear and anxiety disorders. Third, and more important, this should have consequences for the treatment of such disorders. If the disorder is obtained via unconscious processes it may be difficult to treat the disorder by means of a cognitive approach to the treatment. Future studies of the processing behind extinction would be of special interest in this regard. It may be that extinction does not erase all effects of fear learning, a view that is supported by research, revealing that extinction is not destruction of the acquired information, but rather new learning [85]. 

The contingency awareness data in Åsli *et al.* [10] and Åsli and Flaten [9] indicates that the self-report of contingency awareness is influenced by factors such as interference and forgetting. Contingency awareness was significantly greater directly after the conditioning phases in Åsli and Flaten [9] compared to after the startle phases. This underlines the importance of assessing awareness as soon as possible after the conditioning session in order to get reliable measures, as pointed out by Lovibond and Shanks [3]. Ideally, one should measure contingency awareness throughout the conditioning phase itself by assessing level of expectancy of the US during CS presentation. This is however not always possible, such as when short inter stimulus intervals are used. 

Åsli and Flaten [8] showed reflex potentiation as early as 100 ms after CS presentation in the paired compared to the unpaired group. Correlational analyses showed a significant, positive correlation between CRs and noise URs, indicating that potentiation was related to CR magnitude. Furthermore, this implies that the CR was mediated by an automatic and pre-attentive process. This is in agreement with previous studies showing that delay conditioning may occur without awareness, and potentiation at short SOAs may be a method to study conditioned learning without assessing contingency awareness with error prone self-reports. 

The similar effects of startle potentiation in Åsli *et al.* [10] and Åsli and Flaten [9] indicate that noise could be employed in conditioning studies to induce fear and be similar in effect as electrical shock in inducing fear. This is in line with previous research arguing that loud noises can be used to induce fear in rats [86,87]. However, as the noise US could elicit eyeblink CR that could have contributed to the startle reflexes, assessing fear as potentiated startle eyeblinks in the same interval as CRs are present should be avoided. 

Åsli *et al.* [10] and Åsli and Flaten [9] indicate that the task of being aware the CS-US contingency following a 800 ms trace in trace fear conditioning with a tone CS is just as easy as being aware of the CS-US contingency in delay conditioning with a similar, only shorter CS, and identical interstimulus interval. This implies that it could be difficult to keep healthy participants unaware of the CS-US contingency during trace conditioning procedures with short interstimulus intervals. This is in agreement with Lovibond and Shanks [3] who pointed out that it would be problematic to assess the effect of unaware conditioning based on manipulations of contingency awareness in normal participants, as the task of making participants unaware of the CS-US contingencies has proven difficult. Assessing startle potentiation at short intervals following CS onset may be utilized to bypass this problem in manipulating awareness. 

## 4. Conclusions

The conclusions are summarized as follows:

(i) The latency of conditioned fear after delay conditioning is about 30–100 ms [9,10], whereas the latency of conditioned fear after trace conditioning is about 500–1500 ms [9].
(ii) Conditioned UR potentiation following delay conditioning has a latency of more than 50 and no more than 100 ms. Correlational analyses indicate that this UR potentiation is a function of processes underlying CR formation [8].
(iii) Potentiated startle at short latencies (100 ms and less) after CS presentation following delay conditioning [8,9,10] and startle potentiation only at 1500 ms after CS presentation following trace conditioning [9] indicate that startle potentiation at short intervals could be a method to investigate pre-conscious learning effects.
(iv) The short latencies from CS onset to changes in reflexive behavior is seen, and is in line with the notion that unconscious processes are able to influence even complex human cognitive and emotion-related information processing [8,9,10].
